# Developing a Tracheal Rendezvous Procedure for Complete High Subglottic Stenosis

**DOI:** 10.3390/life13030740

**Published:** 2023-03-09

**Authors:** Philip A. Weissbrod, Bharat Panuganti, Jenny Yang, George Cheng

**Affiliations:** 1 Department of Otolaryngology, University of California San Diego, La Jolla, CA 92037, USA; 2 Department of Medicine, Division of Pulmonary, Critical Care, and Sleep Medicine, University of California San Diego, La Jolla, CA 92037, USA

**Keywords:** subglottic stenosis, T-tube, KTP laser, aphonia, airway obstruction, rendezvous

## Abstract

Complete subglottic stenosis is often managed with surgical resection. However, involvement of the high subglottis can limit candidacy for open resection, and there are few treatment options for these patients. We refined an endoscopic approach that evolved into a tracheal rendezvous technique with T-tube placement as an alternative to open surgical resection. Here, we present our series, technique, and outcomes. A retrospective review was performed to identify patients who underwent endoscopic management of complete high subglottic stenosis at the University of California San Diego. The surgical technique was initially a two-step staged procedure and was subsequently revised to a single-stage procedure with stenosis ablation, dilation, and insertion of a T-tube, which was completed in one day. Patients were seen at regular follow-up intervals for reassessment. Five patients were identified with complete stenosis not amenable to surgical resection. The average age of the cohort was 44.8 years. The etiology of stenosis in all patients was related to prolonged intubation and tracheostomy, and the average length of stenosis was 19.6 mm. Stenosis resection was accomplished via laser ablation and balloon dilation, and the average T-tube length was 50.3 mm. All patients were discharged on postoperative day one. Two patients developed airway crusting within the T-tube and required emergency department visits. Decannulation was attempted in three patients, although failed in two. Tracheal rendezvous is a safe and effective procedure for patients with grade IV subglottic stenosis. This provides a feasible endoscopic alternative to patients who are not candidates for open surgical resection, ye are motivated to have phonatory capacity.

## 1. Introduction

Complete subglottic stenosis presents a unique set of compelling challenges. Open surgical resection is the most effective intervention for management and the procedure of choice for many surgeons [1,2]. Cricotracheal resection, or resection of involved trachea along with anterolateral cricoid cartilage with direct anastomosis of the trachea to the thyroid cartilage, is indicated when the stenosis involves the subglottis. Candidacy for cricotracheal resection can be limited by a number of factors including, but not limited to, the proximity of the stenosis to the true vocal folds and cartilaginous involvement or destruction of the posterior cricoid plate. Additionally, some patients are not suitable candidates for major open resection due to medical morbidities including severe uncontrolled diabetes, and cardiac or respiratory illness [3]. Patients who are not candidates for cricotracheal resection, especially those with severe or complete stenosis, have few alternatives to alleviate tracheostomy dependency and aphonia, both of which have a significant impact on quality of life [4,5,6]. Having an alternative management option to major open surgery would be a welcome addition for providers to offer select patients with complete subglottic stenosis.

Here, we present an endoscopic alternative to surgical resection for the complete high subglottic stenosis patient population. In this series of five patients, we have begun to refine a technique to allow for safe transglottic recannulation of grade IV Cotton–Myer stenotic [7] segments via laser ablation, dilation, and postoperative stenting with a T-tube.

Montgomery T-tubes have been used for over half of a century for stenting tracheal and subglottic stenosis [8]. They are distinguished from linear tracheal stents in that the “T” limb allows for ventilation and stabilization, which prevents stent migration, a common complication of straight silicone stents, [9] making T-tubes ideal for cases of high subglottic stenosis. In the most recent case, presented in this series, the T-tube design was personalized to the patient’s anatomy to enhance the fit and reduce potential related complications. In select cases, patients can be decannulated after an extended period once the scar tissue has formed around the tube to create a stable fibrotic lumen.

As this technique has been refined, it has progressed from a two-stage procedure to a one-stage procedure that takes advantage of experience with esophageal rendezvous [10,11], which helps guide the safe reestablishment of an airway and reduces the potential for extraluminal deviation and complications. Here we present our collective experience with this novel endoscopic approach for the management of unresectable grade IV subglottic stenosis.

## 2. Materials and Methods

This study was approved by the Institutional Review Board for exemption at the University of California San Diego, due to the use of deidentified, pre-existing patient data.

### 2.1. Data Collection

A retrospective chart review was performed to identify all patients who underwent treatment for a complete grade IV subglottic stenosis with laser ablation and placement of a T-tube. In addition to details regarding the patients’ surgical procedure, data extracted from the electronic medical record included age, gender, weight, height, body mass index (BMI), American Society of Anesthesiologists (ASA) class, medical and surgical history, medical comorbidities, history pertaining to tracheostomy and duration of stenosis, and outcome pertaining to the airway.

### 2.2. Surgical Technique

All patients underwent general anesthesia and trans-stomal intubation. Four of the five cases were conducted with a Mallinckrodt Endotracheal Tube (Medtronic, Minneapolis, MN, USA) in place. Ventilation in the fifth case, in the series, was managed by placing a Provox Flexiderm laryngectomy baseplate (Atos Medical Inc., New Berlin, WI, USA) over the stoma with a modified endotracheal tube adapter to facilitate the connection to the anesthesia circuit. Following the establishment of ventilation through their tracheotomy, a Dedo laryngoscope (Pilling, Morrisville, NC, USA) was placed transorally and suspended at the glottis. The distance from the superior surface of the vocal fold to the cranial aspect of the stenosis was measured by marking a rigid telescope at the edge of the laryngoscope and measuring the marks with a ruler.

Ablation of the subglottic stenosis was performed with a laser in all cases. Typically, the Dedo was advanced into the subglottis past the glottis to protect the vocal folds during lasering. For the first case, an AcuBlade^TM^ CO_2_ laser (Lumenis Limited, Yokneam, Israel) set at 10 watts (W) and in superpulse mode was used. For all other cases, a 532 nanometer (nm) KTP laser (American Medical Systems, Minnetonka, MN, USA) was used on either 6 W continuous or 40 W with 15 ms pulse width, and 2 pulses per second. In all but the fifth case, the trajectory of the laser energy used to core out the stenosis was based on the expected location, which was extrapolated from the radiographic imaging and clinical judgment. In the fifth case, a flexible bronchoscope was inserted through the tracheotomy and retroflexed, allowing for the bronchoscopic light to serve as a guide for laser ablation of the stenosis. A Kleinsasser straight 1 millimeter (mm) laryngeal probe (Karl Storz, El Segundo, CA, USA) was used intermittently in all cases to probe the apex of the resection and guide the laser ablation until the distal tracheal lumen was entered. 

**Figure 1:**
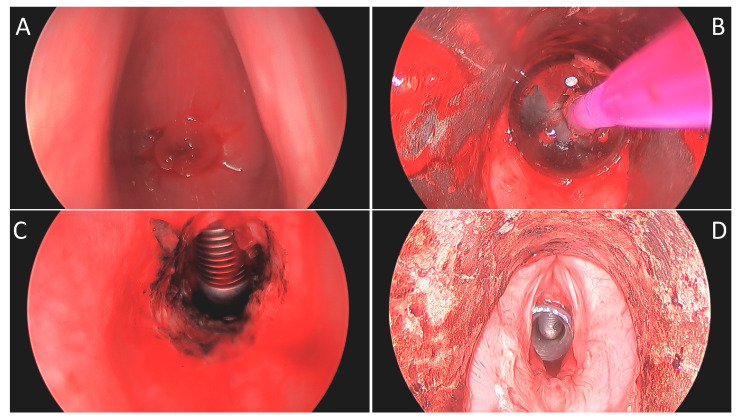
Intraoperative 0° telescopic images of patient “C”: (**A**) View through vocal folds with stenosis apex 16 mm from the superior surface of the vocal fold with anterior extent just inferior to Broyles ligament. (**B**) Following KTP ablation and balloon dilation to 15 mm. (**C**) Post-dilation view of 1.5 cm length stenosis and laser tube below. (**D**) After placement of a 13 mm diameter T-tube with a proximal limb of 11 mm.

Once the distal airway was entered, the residual stenosis was widened circumferentially with additional laser ablation and dilated with CRE balloon dilators (Boston Scientific, Marlborough, MA, USA) to between 12 and 15 mm (Figure 1B). A Montgomery Safe-T-Tube (Boston Medical Products, Shrewsbury, MA) was, then, trimmed appropriately and placed to span the stenotic region, terminating cranially just inferior to the infraglottis (Figure 1D). For the fifth case in the series, a custom T-tube (Hood Medical, Pembroke, MA, USA) was fashioned based on preoperative imaging. Placement of the T-tube was completed by a variety of techniques.

The first two cases were staged procedures. The first stage included coring out the stenosis and dilating the subglottis, with a second planned stage consisting of further dilation and placement of a T-tube. The latter three cases were completed in a single stage with both laser-assisted subglottic recannulation and T-tube placement being accomplished within one sitting.

Patients were admitted for overnight observation in a monitored setting. Patients were discharged on the postoperative day 1 following appropriate stent teaching, including education regarding the necessity of T-tube capping to prevent airway crusting and to reduce the potential for obstruction.

### 2.3. Post-Operative Care

Patients were seen typically at 1, 6, and 12 weeks postoperatively to evaluate for granulation and crusting, and to ensure appropriate positioning of the stent. Subsequent follow-ups occurred, on average, every three months. T-tubes were exchanged intraoperatively either at 1 year or if a biofilm formed and resulted in crusting and/or a local reaction. Attempts at a T-tube free trial (i.e., removal without replacement) were made if the airway looked stable at the time of the exchange.

## 3. Results

Five patients underwent T-tube placement over a 3-year period. Two patients underwent staged procedures, while the remaining three underwent a single procedure for the ablation of their stenosis and the placement of a T-tube. The etiology of stenosis in all patients involved extended periods of intubation and a subsequent tracheostomy. Two patients were intubated following motor vehicle accidents, two for respiratory failure secondary to pneumonia, and one after a suicide attempt. Three of the five (60%) patients were male; the average age of the population at the time of their procedures was 44.8 years, and the average BMI was 28.8 kg/m^2^. Four (80%) patients reported being former or active intermittent tobacco users (Table 1).

**Figure 2:**
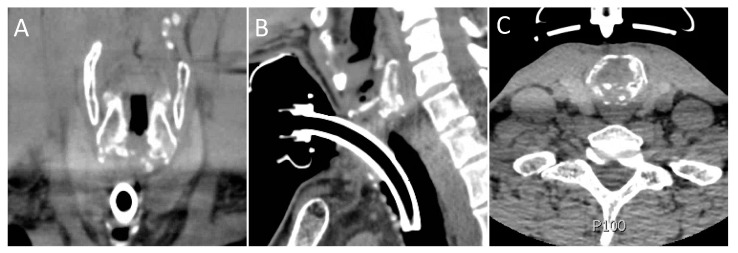
Preoperative coronal (**A**), sagittal (**B**), and axial (**C**) images of patient “A”. Note diffuse sclerosis and destruction of the posterior cricoid plate.

The indication for T-tube placement, as opposed to open resection, in 4 of the 5 (80%) cases was the proximity of stenosis to the vocal folds, combined with sclerosis of the cricoid in 2 of these cases, as well (Figure 2). In the fifth patient, there was concern about patient tolerance of open resection and recovery due to significant cardiac pathology and poor pulmonary status. Two patients (40%) had significant cardiac disease, and one of these patients also had poorly controlled type 2 diabetes mellitus. No patients had an autoimmune disease. The median ASA classification score was three (Table 1).

Tracheal stenosis, as measured in the operating room, started on average 13 mm from the cranial edge of the vocal fold. The length of complete stenosis averaged 19.6 mm. Balloon dilation was performed up to 15 mm in 4 patients and 12 mm in 1 patient. The average T-tube total length was 50.3 mm, with the cranial limb averaging 16.6 mm. The T-tube diameter was 13 mm in 3 patients, 12 mm in 1 patient, and 10 mm in 1 patient (Table 1).

**Figure 3:**
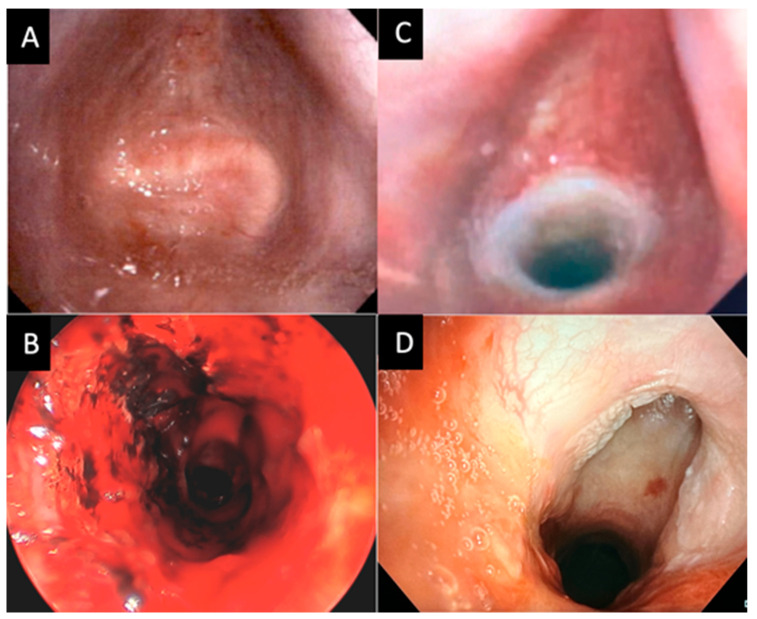
Bronchoscopic images of patient “E”. (**A**) Initial flexible bronchoscopy showing complete fusion and occlusion of the subglottic space. (**B**) Intraoperative image postrecannulation of tracheal stenosis. (**C**) Flexible bronchoscopy at the 2-week follow-up with T-tube in place. (**D**) Flexible bronchoscopy after T-tube decannulation with marked improvement of tracheal stenosis.

Postoperatively, two patients (40%) were treated with antibiotics due to airway colonization. Two patients (40%) required emergency department visits for airway crusting. Of these, one patient (20%) was ultimately deemed incapable of caring for the T-tube, which was removed and replaced with a tracheostomy tube. The other required the removal of crusting on two separate occasions due to non-compliance with the cleaning regimen and capping. Four of the five patients (80%) were noted to be alive at the last chart check, with one patient being deceased for unknown reasons nineteen months after stent placement. Stent decannulation was attempted in three patients (60%); it was successful in one (Figure 3) and failed in the other two patients.

## 4. Discussion

The etiology of subglottic stenosis can be secondary to a number of traumatic processes including intubation, tracheotomy, caustic injury, rheumatologic disease, or chemoradiation. Severe tracheal stenosis can significantly impact the quality of life due to tracheostomy dependency, dyspnea, and/or aphonia. Typically, when tracheal stenosis is more aggressive, more procedures are required to maintain patency, and there is a higher likelihood that open surgical resection is required for definitive management [12].

In a European multicenter study of open tracheal and cricotracheal resection, patients with a higher grade of stenosis with involvement of multiple airway subsites and more comorbidities had higher rates of tracheostomy dependency, longer times to decannulation, lower decannulation rates, and higher complication rates following resection [13]. Postoperative complications of open resection are not trivial. The same European study cited an overall complication rate of 46%, with the most common complications relating to airway edemas (13.3%), dehiscence of the tracheal anastomosis (10.8%), granulation of tissues (7%), and vocal fold paralysis (7%).

Despite the risk profile, cricotracheal stenosis is considered most effectively treated with a single-stage cricotracheal resection [14,15]. However, it is difficult to draw definitive conclusions regarding the optimal management of grade IV stenosis, specifically, as it represents a relatively small proportion of reported study populations, and more granular clinically pertinent details concerning patient presentation (e.g., the precise location of the stenosis), surgical decision-making, and even surgical candidacy are not always available [14,16].

For high complex stenosis involving both the subglottis and glottis, some providers advocate for both open excisions of the stenosis and cartilaginous augmentation of the posterior cricoid and anterior thyroid cartilage. In a series of 20 patients, 6 were treated for multilevel stenosis, as described above, and 5/6 were decannulated by the 2-year mark. All patients in the series required 2 months of solid laryngeal stenting and nearly 22 months of T-tube stenting. Notable reported surgical complications included infection (16%), subcutaneous emphysema (16%), trachea-cutaneous fistula (16%), and dysphonia (16%) [17].

It should be noted that open airway resections under these circumstances are complex and challenging surgical cases. Not all patients are acceptable surgical candidates due to a variety of reasons. Furthermore, those institutions reporting large series with tolerable complication rates typically have extensive experience with this pathology, and replication of their surgical outcomes may be limited in less seasoned centers. As such, having the capacity to provide a procedure that is lower risk, technically more familiar for more providers (surgeons or interventionalists), and that does not eliminate the option for subsequent open surgical resection, may be of interest to many.

A review of the published literature showed sparse case reports on tracheal recannulation and stent placement for high subglottic complete stenosis [18,19]. To our knowledge, this is the largest series reported. Additionally, most cases reported in the literature involve membranous-like complete stenosis and not the more severe stenosis characterized by tracheal collapse, loss of framework, and longer diseased segments. In this series, the average length of stenosis was nearly 2 cm. Prasad et al. presented a case of complete stenosis postcricoid split and rib graft, suggesting that this technique may also be utilized in cases of open surgical failure [19].

Given that there have been a number of these more severe subglottic stenosis cases presented to our institution within a relatively short timeframe, we have taken the opportunity to refine our technique with each successive case. A prime example of this is the condensing of the process into a single-stage operation because of the rapid restenosis we observed within 6 weeks after the first step of the staged procedures. In both staged cases, the stenosis had almost completely returned to grade IV stenosis at the time of the second planned procedure.

Laser choice has also evolved. The first case used a line-of-sight CO_2_ laser, a technique that reduced access to the distal stenosis for ablation due to the limits of the line of site geometry. In subsequent cases, a fiber-based KTP laser was delivered through a handpiece to access the more distal end of stenoses. Set at 6 W continuous, KTP represents an excellent tool to ablate mature stenosis in an expeditious fashion.

One of the most important advances in our technique came with the use of a retroflexed flexible bronchoscope in the distal airway, similar to rendezvous esophageal recannulation. The presence of the scope with illumination introduced via the patient’s tracheal stoma allowed for enhanced guidance for the transoral surgeon and reduced the likelihood of deviating outside of the airway into the paratracheal space or the esophagus when laser-ablating the stenosis.

Finally, T-tube design has also evolved. In the first four patients, a standard off-the-shelf T-tube was used and modified intraoperatively. For the fifth case in the series, a custom T-tube was designed based on patient-specific characteristics derived from preoperative imaging. The T-tube was crafted with a personalized angle at the junction of the external and the proximal limb. The benefit of customized tubes, aside from lacking the need for intraoperative manipulation and assurances of smooth proximal and distal ends, is the reduced sequential endoscopic management typically required after placement [20]. When angled and seated appropriately within the airway, the tube is less likely to create irritation and subsequent granulation.

Drawing conclusions about the quality and effectiveness of a procedure from a small sample size has inherent limitations. Nonetheless, all patients were safely managed without any significant complications. It should be highlighted that the use of a T-tube should be reserved for patients capable of reliable daily care, a limitation that manifested in one of the patients in the series who was medically fragile and ill-suited to perform the requisite care and hygiene.

To date, only one patient in this series has been able to tolerate T-tube removal. This likely speaks to the severity and location of the stenosis, and gross disruption of the laryngotracheal framework. This also highlights the notion that T-tubes require regular maintenance and at least, the annual exchange. Similar to dilation procedures for advanced stenosis, routine T-tube care may be costlier in the long term than tracheal resection, a factor that should at least be considered when selecting candidates for this procedure [21]. Nonetheless, the data presented herein should reassure providers considering endoscopic management of high, complete laryngotracheal stenosis with the placement of the T-tube, as long as patient-centric factors (e.g., the capability of self-care) are carefully assessed and the need for serial procedures is directly communicated.

## 5. Conclusions

Overall, tracheal recannulation and rendezvous, in this small series, was shown to be a safe procedure with good outcomes and should be considered as an option for patients with high tracheal stenosis, who may not be good surgical candidates for cricotracheal or tracheal resection. The procedure is technically straightforward and may be more easily adapted to the practice of endoscopic surgeons or interventionalists, who are familiar with endoscopic dilation techniques and T-tube management.

## Figures and Tables

**Table 1:** Baseline characteristics and outcomes of the study population.

	Age	Gender	BMI (kg/m^2^)	Tobacco Use History	ASA Class	Comorbidities	Maximal Dilation Diameter (mm)	T-Tube Diameter (mm)	T-Tube Proximal Limb Length (mm)	T-Tube Distal Limb Length (mm)	Stenosis Distance from Vocal Folds (mm)	Stenosis Total Length (mm)	Laser	Outcome Summary
Patient A	39	M	21	Former smoker	3	Seizure disorder	15	13	20	25	<10	22	CO_2_	Alive. T-tube decannulation not attempted.
Patient B	62	M	37.4	Former smoker	3	Hypertension, coronary artery disease, type 2 diabetes, COPD	15	12	17	24	10	25	KTP	Deceased (unknown etiology).
Patient C	26	M	28	Never smoker	3	None	15	13	11	16	16	15	KTP	Alive. Failed attempt at T-tube removal. Currently with T-tube
Patient D	69	F	34.8	Former smoker	4	Congestive heart failure, pacemaker, hypothyroidism	15	13	15	17.5	10	15	KTP	Alive. Failed attempt at T-tube removal. Currently with tracheostomy tube.
Patient E	28	F	23	Active, intermittent smoker	2	Polysubstance abuse	12	10	20	25	17	21	KTP	Alive. T-tube decannulation successful 18 months postinsertion.

## Data Availability

Not applicable.
